# Problems with accessing healthcare and associated factors among reproductive-age women in Somaliland: a multilevel analysis of data from the 2020 SLDHS

**DOI:** 10.3389/fgwh.2026.1644372

**Published:** 2026-03-10

**Authors:** Hodo Abdikarim, Yahye Dayib Aw-Ali, Awale Ali Omer, Abdisalam Hassan Muse

**Affiliations:** 1 Faculty of Science and Humanities, School of Postgraduate Studies and Research (SPGSR), Amoud University, Borama, Somalia; 2 Gabiley Mental Hospital, Gabiley, Somalia; 3 Research and Innovation Center, Amoud University, Borama, Somalia

**Keywords:** healthcare access, maternal mortality, multilevel analysis, problems, reproductive women, Somaliland

## Abstract

**Background:**

Access to healthcare, particularly for women of reproductive age, is critical for achieving Universal Health Coverage (UHC) and reducing maternal mortality. Somaliland faces significant challenges in healthcare access, but specific barriers for women of reproductive age remain understudied. This study aimed to evaluate healthcare access problems faced by this population in Somaliland.

**Methods:**

The study utilized secondary data sourced from the Somaliland Demographic and Health Survey. The outcome of the study was barriers to healthcare access. A two-level mixed-effects logistic regression approach, along with 95% confidence intervals (CIs), was employed to determine factors related to healthcare issues among women of reproductive age. Statistical significance was declared for p-values below 0.05.

**Results:**

Nearly 75% of women reported at least one barrier to healthcare access**.** The multilevel analysis revealed that being in the age group 25-29 (AOR = 1.58; 95% CI; 1.00–2.48), 35–39 (AOR = 1.86; 95% CI; 1.12–3.07), 40–44 (AOR = 1.84; 95% CI; 1.03–3.29), a secondary education level (AOR: 1.70; 95% CI; 1.13–2.560), higher education (AOR = 3.72; 95% CI; 1.96–7.05), women with employed husbands (AOR = 0.69; 95% CI: 0.57–0.84) non-users or those who intend to use later (AOR = 0.51: 95% CI; 0.30–0.85), and having five and more children (AOR = 0.78 95% CI; 0.63–0.96) were significantly associated with healthcare problems at an individual level. On the contrary, regions Woqooyi-galbeed (AOR = 0.50 95% CI; 0.35–0.72), Togdheer (AOR = 0.41; 95% CI; 0.28–0.60), Sool (AOR = 0.37; 95% CI; 0.25–0.54), and Sanaag (AOR = 0.54; 95% CI; 0.38–0.76), women in the middle wealth status (AOR = 2.26; 95% CI; 1.55–3.32), fourth wealth index (AOR = 3.14; 95% CI; 2.17–4.56), and the highest wealth status (AOR = 4.23; 95% CI; 2.88–6.22) were the community-level determinants in access to healthcare.

**Conclusion:**

A substantial proportion of women of reproductive age in Somaliland experience significant barriers to healthcare access. Addressing these challenges requires targeted interventions focusing on improving the socioeconomic status, infrastructure, accessible and affordable healthcare services, and region-specific strategies.

## Introduction

Accessible, available, affordable, and culturally appropriate healthcare services are essential for all individuals, and particularly crucial for vulnerable populations such as pregnant women and their infants, who have specific healthcare requirements (1, 2). The understanding of “access” is more effectively communicated through three interconnected dimensions: availability, affordability, and acceptability (3). In essence, healthcare access exists when services are sufficiently available and when individuals have a realistic opportunity to receive that care. However, barriers to accessing these services can arise from a variety of sources, including financial, organizational, social, or cultural strains within the community (4). Access to comprehensive and high-quality healthcare is vital for promoting good health, decreasing the likelihood of disabilities, preventing and managing illnesses, and advancing health equity (5).

The international health community is aiming for an ambitious goal of achieving Universal Health Coverage (UHC) by 2030, but progress in health service coverage has shown no improvement since 2015. Specifically, in 2021, approximately 4.5 billion individuals lacked complete access to necessary health services. Adding to this challenge, the financial impact of healthcare remains substantial, with an estimated 2 billion people facing catastrophic health expenditures or impoverishment due to out-of-pocket costs in 2019 (6). These issues directly undermine the objectives outlined in Sustainable Development Goal-3 (SDG-3), particularly targets 3.8 and 3.7. These targets highlight the importance of achieving UHC and ensuring access to sexual and reproductive health services – including family planning information and education – as well as the incorporation of reproductive healthcare into national strategies and initiatives by 2030 (6, 7). Indeed, the consequences of inadequate healthcare access are far-reaching: globally, about 400 million individuals face challenges in accessing healthcare services, resulting in eight million deaths from preventable health conditions annually. This issue is especially severe in low- and middle-income nations, leading to a significant economic impact estimated at approximately six trillion USD (8).

The World Health Organization and the World Bank report that approximately 50% of the world's population remains unable to obtain essential health services. This lack of access significantly contributes to poverty, with healthcare expenditures driving 100 million individuals into extreme poverty annually (9). This situation is particularly acute in sub-Saharan Africa, where access to healthcare stands at just 42.56% (10), demonstrating a significantly low level of coverage. Studies have identified numerous interconnected factors that limit access to healthcare, including financial constraints, geographical barriers, the mother's education level, health insurance coverage, the husband's education, a woman's marital status, place of residence, stigma-related matters, and the number of children in the family (11–14).

Reflecting these broader trends, maternal health challenges persist as a major issue and represent ongoing priorities from the MDGs for economically disadvantaged nations like those in Africa (15, 16). In 2015, the Sub-Saharan region experienced a significant impact, with a mortality rate of 546 per 100,000 live births (14, 17). As previous studies indicated, maternal and child mortality a consequences of insufficient utilization of healthcare services in countries (18). While the proportion of women receiving four or more antenatal care (ANC) services in developing regions averages 52%. However, sub-Saharan Africa lags, with only 49% of women receiving this level of care (19). East Africa has demonstrated notable advancements in reducing maternal mortality, with a 48% decline in the maternal mortality ratio since 2000. However, notable disparities within the region still exist. The variation in maternal mortality ratios, from a low (3 deaths per 100,000 live births) in Seychelles to a high (1,223 deaths per 100,000) in South Sudan, highlights the ongoing challenges in achieving equitable maternal health outcomes across the region (20, 21). Consequently, women in sub-Saharan Africa are not utilizing fundamental healthcare services to the necessary extent, leading to inadequate coverage of maternal health services and impacting the well-being and survival of both mothers and infants (22).

Somaliland, a nation with limited financial resources, faces challenges related to both infectious and non-infectious illnesses, as well as issues concerning maternal, neonatal health, and nutrition (23). According to the 2020 Somaliland demographic and health survey, a substantial number of women (67.9%) encounter at least one barrier when seeking healthcare. Among these challenges, women reported financial constraints as their primary obstacles to healthcare access (61.1%), and distance to health facility as a major issue for women (58%) (24). Moreover, the healthcare system in Somaliland encounters a range of obstacles, including inadequate investment in health, limited technical proficiency in research design, data gathering, and analysis, as well as challenges in disseminating and effectively utilizing evidence-based research (25).

Due to medical complications, obstetric hemorrhage, infections related to pregnancy, and hypertensive conditions, Somaliland has a high maternal mortality rate, where approximately 396 maternal deaths occur per 100,000 live births, ranking highly on a global scale but below certain comparable countries (23, 26). Improving health research systems enhances comprehension of population health requirements through the recognition of socio-cultural obstacles to accessing health services, discrepancies between the community and the present healthcare system influencing healthcare provision, and barriers within the system (27, 28).

Recognizing this need, according to our understanding, no published studies are available concerning the obstacles to accessing healthcare among women of reproductive age in Somaliland. This study, therefore, aims to bridge this critical knowledge gap by rigorously examining the obstacles to healthcare access faced by women of reproductive age in Somaliland. Utilizing data from the nationally representative 2020 Somaliland Demographic and Health Survey (SLDHS), this research seeks to identify the key barriers and associated factors that impede women's access to essential healthcare services. Through this in-depth analysis, the study intends to provide actionable insights for policymakers and healthcare providers, informing the development of targeted interventions that can improve healthcare access, reduce maternal mortality, and ultimately contribute to achieving Universal Health Coverage (UHC) in Somaliland.

## Methods

### Study area

The current study was conducted in Somaliland. It is an unrecognized country on the southern coast of the Gulf of Aden and is bordered by Djibouti to the northwest, Ethiopia to the southwest, and Somalia to the east. The country has five main regions namely Awdal, Woqooyi galbeed, Togdheer, Sanaag, and Sool with an area of 176,120 KM^2^. The population is estimated around 4.2 million who are Somali ethnic and Muslims. Agriculture and livestock production is the main source of income of the population (29).

### Data source, study design, and sampling procedure

We used data from the 2020 Somaliland Demographic and Health Survey. It aims to provide up-to-date information to monitor the health situation in the country. The SLDHS is a nationally representative survey that aims to collect data on many health-related topics, including healthcare access, fertility levels, maternal and child health, gender, nutrition, HIV/AIDS, housing conditions, family planning, gender-based violence, female circumcision, and chronic diseases (30–36).

A robust, multi-stage stratified cluster sampling technique was used by the SLHDS 2020 to capture data across various communities. The sampling strategy was tailored to the geographic context, with urban and rural areas following a three-stage stratified cluster approach, and nomadic regions utilizing a two-stage design. Primary sampling units (PSUs) and secondary sampling units (SSUs) were identified using probability proportionate to size (PPS) based on dwelling structures and household lists, respectively. Households were systematically selected as the ultimate sampling units (USUs), ensuring a robust and reliable dataset for analysis (24).

For this current study was first geographically restricted to Somaliland. Within this target area, we identified 11,286 women of reproductive age (15–49) who met the initial eligibility criteria. However, 7536 women were subsequently removed due to incomplete responses regarding the outcome variable (barriers to healthcare access) or missing exposure variables data. This resulted in a final analytical sample of 3,750 women.

### Study variables

#### Outcome variable

The primary focus of the current study was barriers to accessing healthcare. This variable was evaluated through asking women binary questions (yes/no) in the DHS survey about four potential barriers: financial constraints, distance to healthcare facilities, treatment permissions, and the need for companionship. A woman was considered to have a barrier to healthcare access (coded as “1”) if she reported experiencing one or more of these issues. Otherwise, she was considered to have no barrier (coded as “0”).

#### Explanatory variables

The explanatory variables were selected based on a previous study that highlighted the potential connection between individual and community-level variables and the challenges associated with accessing healthcare services (10, 37, 38). The age of the women, education, employment status of women, husband's employment, sex of the household head, total children ever born, contraceptive use and intention, and place of delivery were individual-level variables. Furthermore, community variables such as the type of place of residence, region, wealth index, and mass media exposure were included in the study.

### Statistical analysis

Descriptive statistics, including frequencies and percentages, were utilized to summarize sample characteristics and healthcare access challenges. Bivariate analyses were conducted using Chi-square tests. To prevent the exclusion of potential confounding variables, a *p*-value cut-off of <0.20 was used as the screening criterion to select candidate variables for the multivariable analysis. Multicollinearity was assessed, yielding a mean VIF of 1.25. Consequently, all eligible variables were entered into a multilevel binary logistic regression analysis incorporating fixed and random effects. Statistical significance in the final model was set at *p* < 0.05.

The fixed effects results of the models were presented as Adjusted Odds Ratios (AORs) with 95% confidence intervals (95% CI). Alternatively, the intra-class correlation (ICC) was used to assess the random effects. Four distinct models were analyzed: Model 0 (empty model) was constructed without any explanatory variables. The second model (model I) utilized individual-level variables, while Model II incorporated community-level factors. The final model (model III) integrated all the explanatory variables to examine their collective impact on problems in accessing healthcare. The Akaike information criterion (AIC) and Bayesian information criterion (BIC) tests were used for the model comparison.

## Results

### Socio-Demographic characteristics of participants

The sociodemographic characteristics of the population were presented in Table 1, about 30.85% were in the age group 25–29, three-fourths (83.36%) had no formal education, almost all (99.07%) of women were unemployed, and a slight majority of their husbands had jobs (51.01%). Slightly more than one-quarter of the participants were in the poorest wealth status (26.45%). Nearly half (41.79%) belong to nomadic, followed by rural residents (30.16%). In regions, (23.71%) of participants were from the Sanaag region, and most families were headed by males (68.88%).

**Table 1:** Socio-demographic and characteristics among reproductive women aged 15–49 years in Somaliland, based on SLDHS 2020.

Characteristics	Frequency	Percentage
Age		
15–19	151	4.03
20–24	738	19.68
25–29	1,157	30.85
30–34	805	21.47
35–39	627	16.72
40–44	215	5.73
45–49	57	1.52
Educational level		
No education	3,126	83.36
Primary	432	11.52
Secondary	137	3.65
Higher	55	1.47
Maternal employment status		
Yes	35	0.93
No	3,715	99.07
Husband/partner employment status		
Yes	1,913	51.01
No	1,837	48.99
Wealth index		
Lowest	992	26.45
Second	507	13.52
Middle	544	14.51
Fourth	779	20.77
Highest	928	24.75
Residence		
Rural	1,131	30.16
Urban	1,052	28.05
Nomadic	1,567	41.79
Region		
Awdal	499	13.31
Woqooyi Galbeed	743	19.81
Togdheer	754	20.11
Sool	865	23.07
Sanaag	889	23.71
Sex of the household head		
Male	2,583	68.88
Female	1,167	31.12
Contraceptive use and intention		
Using modern method	114	3.04
Using traditional method	4	0.11
Non-user, intends to use later	370	9.87
Do not intend to use	3,262	86.99
Place of delivery		
Home	2,600	69.33
Health institution	1,150	30.67
Total children ever born		
Less than five	1,826	48.69
Five and more than five	1,924	51.31
Mass media exposure		
No	2,814	75.04
Yes	936	24.96

Regarding contraceptive use, (86.99%) did not use contraceptives, while (3.04%) used the modern method. Approximately (69.33%) delivered at home, and the vast majority (75.04%) had no media exposure.

### Prevalence of problems in healthcare access in Somaliland

**Figure 1:**
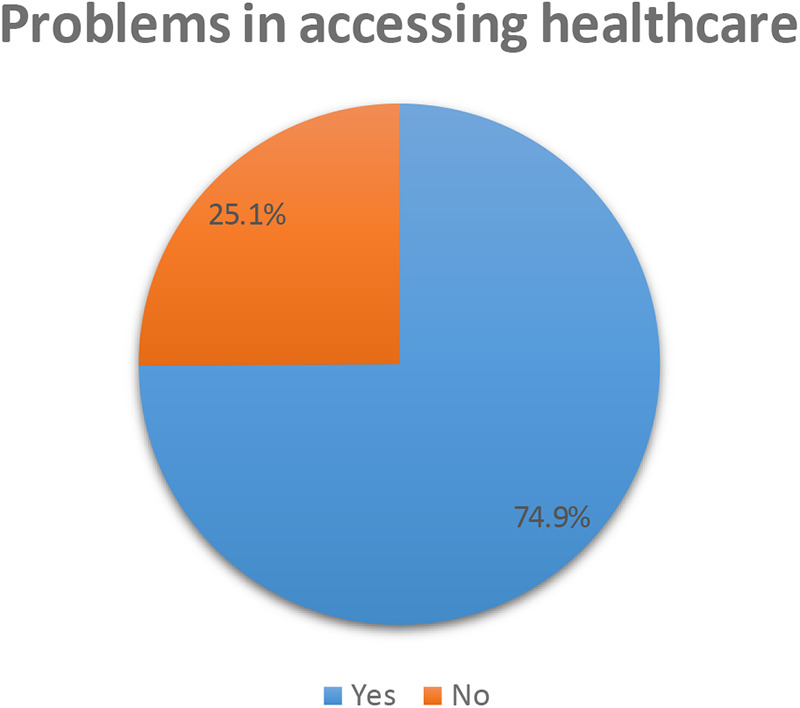
Prevalence of problems in accessing healthcare among reproductive age women in Somaliland, insights from the 2020 SLDHS.

 Figure 1 shows barriers to healthcare access in Somaliland were significant, with a magnitude of 74.9% (95% CI: 735–.763). Among women of reproductive age, the most prevalent obstacles to accessing healthcare included financial constraints at 69.41% and the distance to health facilities at 66%.

#### Bivariate analysis using chi square test

 Table 2 presents the results of bivariate analysis of barriers to healthcare access among reproductive-age women in Somaliland. As shown in the table, all twelve variables were significantly associated with accessing care using the chi-square test. Age of the mother was significantly associated with healthcare access problems (X^2^ = 17.3351, *p* = 0.008). Maternal education also showed a strong association (X^2^ = 111.3337, *p* = 0.000), with women who had no formal education reporting a substantially higher proportion of healthcare access problems (X^2^ = 111.3337, *p* = 0.000). Furthermore, husband/partner employment status was significantly linked to healthcare access challenges (X^2^ = 91.0328, *p* = 0.000), indicating that women whose husbands/partners were unemployed faced greater barriers to accessing healthcare.

**Table 2:** Bivariate analysis of barriers to healthcare access in Somaliland based on SLDHS 2019–2020.

Characteristics	Healthcare access problem		Chi-square	Df	*P*-value
	Yes	No			
Age in 5-year groups					
15–19	117 (77.48)	34 (22.52)	17.3351	6	0.008
20–24	538 (72.90)	200 (27.10)			
25–29	836 (72.26)	321 (27.74)			
30–34	640 (79.50)	165 (20.50)			
35–39	473 (75.44)	154 (24.56)			
40–44	161 (74.88)	54 (25.12)			
45–49	47 (82.46)	10 (17.54)			
Maternal education					
No education	2,433 (77.83)	693 (22.17)	111.3337	3	0.000
Primary	286 (66.20)	146 (33.80)			
Secondary	74 (54.01)	63 (45.99)			
Higher	19 (34.55)	36 (65.45)			
Maternal occupation status					
Yes	30 (85.71)	5 (14.29)	2.1677	1	0.141
No	2,782 (74.89)	933 (25.11)			
Husband/partner employment status					
Yes	1,308 (68.37)	605 (31.63)	91.0328	1	0.000
No	1,504 (81.87)	333 (18.13)			
Wealth quantile					
Lowest	864 (87.10)	128 (12.90)	208.1951	4	0.000
Second	420 (82.84)	87 (17.16)			
Middle	418 (76.84)	126 (23.16)			
Fourth	549 (70.47)	230 (29.53)			
Fifth	561 (60.45)	367 (39.55)			
Residence					
Rural	869 (76.83)	262 (23.17)	14.2551	2	0.001
Urban	817 (77.66)	235 (22.34)			
Nomadic	1,126 (71.86)	441 (28.14)			
Region					
Awdal	339 (67.94)	160 (32.06)	23.2386	4	0.000
Woqooyi/gabeed	579 (77.93)	164 (22.07)			
Togdheer	577 (76.53)	177 (23.47)			
Sool	671 (77.57)	194 (22.43)			
Sanaag	646 (72.67)	243 (27.33)			
Sex of the household head					
Male	1,917 (74.22)	666 (25.78)	2.6280	1	0.105
Female	895 (76.69)	272 (23.31)			
Contraceptive use and intention					
Use modern method	71 (62.28)	43 (37.72)	21.8740	3	0.000
Using traditional method	1 (25.00)	3 (75.00)			
Non-user, intends to use later	299 (80.81)	71 (19.19)			
Do not intend to use	2,441 (74.83)	821 (25.17)			
Place of delivery					
Home	2,030 (78.08)	570 (21.92)	43.1658	1	0.000
Health institution	782 (68.00)	368 (32.00)			
Total children ever born					
Less than five	1,322 (72.40)	504 (27.60)			
Five and more than five	1,490 (77.44)	434 (22.56)	12.7085	1	0.000
Mass media exposure					
No	2,202 (78.25)	612 (21.75)	64.0726	1	0.000
Yes	610(65.17)	326 (34.83)			

Wealth status also exhibited a significant association (Chi-square = 208.1951, *p* = 0.000), implying that women from lower wealth quantiles experienced more healthcare access problems compared to those from higher quantiles. Place of residence was another significant factor (X^2^ = 14.2551, *p* = 0.001), suggesting that rural, urban, and nomadic women have different experiences regarding healthcare access. Regional disparities also played a role, with a significant association observed between region and healthcare access problems (X^2^ = 23.2386, *p* = 0.000).

Moreover, contraceptive use and intentions showed a significant relationship with healthcare access (X^2^ = 21.8740, *p* = 0.000), indicating that the healthcare access challenges were more prevalent (80%) among non-users of contraceptives. Similarly, place of delivery was significantly associated with healthcare access challenges (X^2^ = 43.1658, *p* = 0.000), suggesting that women who delivered at home experienced different barriers compared to those who delivered in a health institution. The total number of children ever born also exhibited a significant association (X^2^ = 12.7085, *p* = 0.000), with women having more children facing greater healthcare access problems. Mass media exposure showed a significant relationship with healthcare access (X^2^ = 64.0726, *p* = 0.000), indicating that women with limited mass media exposure faced greater barriers.

### Multilevel binary logistic regression results

 Table 3 illustrates the results of multilevel binary logistic regression analysis of problems in healthcare access among women in Somalia. Findings from the multilevel binary logistic regression analysis demonstrated both individual and community-level variables were associated with problems of healthcare access. The study revealed that women aged 35–39 were 1.86 times more likely to have healthcare access problems compared to those aged 25–29 (AOR:1.58 CI95%, 1.00,2.48). In addition, low-educated women are more likely to experience healthcare access challenges compared to those with higher levels, with an AOR of 3.72 (95% CI: 1.96–7.05) indicating that these challenges are more prevalent among low-educated women. Women from Sanaag region were significantly less likely to face healthcare access problems than those from Awdal (reference), with odds reduced by approximately 46% (AOR: 0.54, 95% CI: 0.38–0.76).

**Table 3:** Multilevel binary logistic regression analysis results for problems in healthcare access and its associated factors among reproductive-age women in Somaliland based on the 2020 SLDHS.

Variables and Category	Model 0 Empty model	Model I Individual-level variables	Model II Community level variables	Model III Both individual and community-level variables
		AOR (95% CI)	AOR (95% CI)	AOR (95% CI)
Age in five-year group				
15–19		Ref		Ref
20–24		1.39 (0.88, 2.20)		1.52 (0.96–2.42)
25–29		1.52 (0.97, 2.39)		**1.58** (**1.00**–2.48)*
30–34		1.12 (0.70, 1.81)		1.13 (0.70–1.84)
35–39		1.76 (1.07, 2.88)*		**1.86** (**1.12**–3.07)*
40–44		1.80 (1.02, 3.18)*		**1.84** (**1.03**–3.29)*
45–49		1.36 (0.58, 3.21)		1.41 (0.59–3.37)
Education level of the mother				
No formal education		Ref		Ref
Primary school		1.32 (1.03, 1.70)*		1.13 (0.87–1.46)
Secondary school		2.18 (1.47, 3.25)*		**1.70** (**1.13**–2.560)*
Higher		4.58 (2.41, 8.71)*		**3.72** (**1.96**–7.05)***
Maternal occupation status				
Yes		Ref		Ref
No		2.67 (0.96, 7.42)		2.38 (0.84–6.68)
Husband/partner's occupation status				
Yes		Ref		Ref
No		.56 (0.47, 0.67)*		**0.69** (**0.57**–0.84)***
Region				
Awdal			Ref	Ref
Woqooyi-galbeed			0.50 (0.35, 0.70)*	**0.50** (**0.35**–0.72)***
Togdheer			0.40 (0.28, 0.58)*	**0.41** (**0.28**–0.60)***
Sool			0.36 (0.25, 0.53)*	**0.37** (**0.25**–0.54)***
Sanaag			0.53 (0.38, 0.75)*	**0.54** (**0.38**–0.76)***
Residence				
Rural			Ref	Ref
Urban			0.93 (0.71, 1.20)	0.88 (0.68–1.15)
Nomadic			0.82 (0.63, 1.06)	0.76 (0.57–1.00)
Wealth index				
Lower			Ref	Ref
Second			1.15 (0.79, 1.66)	1.12 (0.77–1.63)
Middle			2.34 (1.62, 3.37)*	**2.26** (**1.55**–3.32)***
Fourth			3.47 (2.46, 4.90*	**3.14** (**2.17**–4.56)***
Highest			4.87 (3.44, 6.89)*	**4.23** (**2.88**–6.22)***
Sex of the house hold head				
Male		Ref		Ref
Female		0.99 (0.83, 1.19)		1.05 (0.87-1.27)
Contraceptive use and intention				
Using modern method		Ref		Ref
Using traditional method		8.30 (0.70, 97.6)		8.89 (0.78–100.4)
Non-User, intends to use later		0.44 (0.26, 0.74)*		**0.51** (**0.30**–0.85)*
Does not intend to use		0.84 (0.54, 1.31)		1.01 (0.65–1.58)
Place of delivery				
Home		Ref		Ref
Health institution		1.13 (0.93, 1.37)		0.88 (0.71–1.08)
Total children ever born				
Less than 5 children		Ref		Ref
5 and more		0.77 (0.62, 0.95)*		**0.78** (**0.63**–0.96)*
Mass media exposure				
No		Ref		Ref
Yes			1.19 (0.96, 1.46)	1.13 (0.91–1.41)
Assessment criteria				
	Model 0	Model I	Model II	Model III
ICC	0.21	0.16	0.13	0.14
AIC	4,043	3,936	3,906	3,850
BIC	4,055	4,054	3,987	4,037
Log-likelihood	−2,019	−1,949.	−1,940	−1,895

Ref, reference; ICC, Intraclass Correlation Coefficient; AIC, Akaike information criterion; BIC, Bayesian information criterion; Bold entries have significant values.

* *P* < 0.05,

** *P* < 0.01,

*** *P* < 0.0001.

Moreover, women with unemployed husbands were less likely to have difficulties in accessing healthcare compared to those with employed husbands, with an AOR of 0.69 (95% CI: 0.57–0.84). Compared to women in the lowest wealth quintile, women in the highest wealth quintile were significantly more likely to experience difficulties accessing healthcare, as indicated by an adjusted odds ratio of 2.26 (95% CI: 1.55–3.32).

Women in the highest wealth quintile were significantly more likely to report healthcare access problems than those in the lowest wealth quintile, demonstrating an odds ratio of 4.23 (95% CI: 2.88–6.22). This suggests that despite their higher socioeconomic position, women in the wealthiest category are substantially more likely to face barriers to healthcare utilization. Non-use of contraceptives was associated with a significantly reduced odds of reporting healthcare access obstacles compared to the use of modern contraceptives, with an odds ratio of 0.51 (95% CI: 0.30–0.85). This indicates that women who do not use contraceptives are less likely to experience barriers in accessing healthcare. Lastly, higher parity (five or more children) was associated with significantly lower odds of experiencing healthcare access problems compared to lower parity (less than five children), with an odds ratio of 0.78 (95% CI: 0.63–0.96).

## Discussion

This study, conducted in Somaliland, investigated the problems women of reproductive age face in accessing healthcare. Utilizing data from the 2020 Somaliland Demographic Health Survey, the research revealed that approximately 75% of women reported experiencing difficulties in accessing healthcare for at least one of four reasons: financial constraints, distance to healthcare facilities, the need for treatment permissions, and the requirement for a companion. The most frequently cited obstacles were financial constraints (69.41%) and geographic distance from health facilities (66.01%).

The current study's magnitude was substantially less than the 93.6% reported by the Ethiopian demographic and health survey (EDHS) in 2011 (39). However, despite international attempts to provide universal health access for all people, the results of the current study indicated a greater magnitude of perceived barriers. Furthermore, this result was higher than those reported in the studies from Tanzania 65% in 2018 (40), 64.5% in South Africa (41), 64% in Rwanda (42), and 51% in Ghana (43), and 45% in Gambia (11). This can be explained by the sociocultural and economic differences between nations, which may influence on how people seek health care (44). While universal health facility coverage is a fundamental requirement for all populations, significant disparities in access and utilization of healthcare services persist among women of reproductive age. These inequities pose a substantial challenge to achieving the Sustainable Development Goals (SDGs), particularly those focused on equitable service provision and universal access to reproductive health care.

Based on the comparison of the four models, Model III, which includes both individual and community-level variables provide the best overall fit according to multiple criteria: Model III has the lowest Akaike Information Criterion (AIC = 3850), along with the highest log-likelihood value (-1,895). According to Model III, this study has demonstrated that healthcare access barriers in Somaliland among women of reproductive age are determined by several individual and community-level factors, including region, age, wealth quantile, education, husband's employment, contraceptive use, and number of children ever born.

Healthcare access for women in Somaliland is significantly determined by a complex interplay of factors, including education, spousal employment, and household wealth. We found that age and healthcare accessibility barriers were correlated, with women 25–29, 35–39, and 40–44 years old having a higher likelihood of reporting healthcare access difficulties than women 15–19 years old. This result is comparable to research conducted in Malaysia and Nigeria (45, 46). A possible explanation for this disparity is that older women are more susceptible to geographical barriers, particularly the long distances required to reach healthcare facilities in peripheral regions. Furthermore, compared to younger cohorts, older women may experience higher levels of financial instability and economic dependency, further limiting their ability to access care.

We further found that women with higher levels of education are linked to higher odds of healthcare access problems compared to those with lower levels of education. Higher levels of education can lead to greater awareness and improved health-seeking behavior in women. This is a consistent study established in Bangladesh (12) and Ethiopia (13). Additionally, we found that access issues to healthcare were substantially correlated with the occupation of the husband. To be more precise, we discovered that women whose husbands were unemployed were less likely to experience difficulties in obtaining healthcare services. This is consistent with a study in Lalitpur, Nepal (47).

The wealth index also affects access to healthcare for Somaliland women of reproductive age, where households with the highest wealth status face greater challenges in accessing healthcare compared to those with the lowest household wealth status. This could reflect higher expectations for the convenience of care, or greater awareness of existing shortcomings in the healthcare system. Consistent with findings from studies in Ethiopia (48) and Sub-Saharan Africa (10). This finding may be driven by the healthcare-seeking behaviors of different economic groups. Wealthier women may have less decision-making autonomy or require male accompaniment to seek care, creating a social barrier to access despite their financial advantage.

Additionally, the region in which respondents reside is strongly associated with the barriers reproductive-aged women encounter in accessing health services. Accessing healthcare is more difficult for women in all regions of Somaliland, like Marodijeh, Togdheer, Sanaag, and Sool, compared to the reference group, which is Awdal. This result is consistent with another study done in Gambia (11).

The study also found that women who were multipara or had ever given birth to more than 4 were less likely to face barriers to accessing medical care. Our results corroborate evidence from prior investigations (49, 50). A possible explanation for this finding is that high parity often correlates with increased female autonomy and decision-making power within the household hierarchy. Unlike younger, low-parity women who may require permission to seek care, multiparous women usually possess the social status and independence to mobilize resources and travel to health facilities, thereby reporting significantly fewer access barriers.

This study, utilizing the first-ever Somaliland Demographic Health Survey (2020), provides crucial baseline insights into the barriers reproductive-aged women face in accessing healthcare within Somaliland. It establishes a vital benchmark against which future progress can be measured, highlighting the significant challenges related to financial constraints and distance to facilities. Regionally within Somaliland, it underscores the urgent need to address equitable service distribution and universal reproductive healthcare access, particularly given the absence of comprehensive prior data in the country. The identification of key factors such as age, education, husband's employment, wealth, parity, and region can inform targeted interventions and policies to improve healthcare access in Somaliland, providing a foundation for evidence-based policy development specifically tailored to the country's unique context.

While this study offers valuable insights, certain limitations warrant consideration. First, the cross-sectional nature of the data limits the ability to establish causal relationships between the identified factors and healthcare access barriers. Second, the reliance on self-reported data may be subject to recall bias and social desirability bias, potentially affecting the accuracy of responses, particularly regarding sensitive topics like contraceptive use and reproductive history. Third, while the study identifies significant associations, it does not explore the underlying mechanisms through which these factors influence healthcare access. Finally, while the multilevel modeling approach accounts for community-level variations, unmeasured contextual factors may still influence healthcare access barriers, and the generalizability of the findings to other settings may be limited due to the unique socio-cultural and economic context of Somaliland.

## Conclusion and policy implications

This study reveals that approximately 75% of reproductive-aged women experience significant barriers to accessing healthcare services. Key factors associated with the difficulties in healthcare access include financial constraints, maternal age, level of education, and socioeconomic disparities. Furthermore, the region in which a woman resides, her husband's employment status, contraceptive practices, and the number of children she has borne also significantly influence her access to healthcare.

These findings necessitate targeted interventions and policy measures tailored to the specific needs of women within different regions and socioeconomic contexts in Somaliland. To achieve equitable access to healthcare, policies must address individual-level factors while also considering the underlying community and regional forces at play.

This requires a multifaceted approach, including prioritizing increased government investment in the healthcare sector, with a specific focus on reproductive health services and infrastructure. It also requires addressing socioeconomic disparities by implementing targeted subsidies and social protection programs to alleviate financial constraints for the most vulnerable women. Furthermore, expanding and strengthening community-based services by deploying mobile health clinics and empowering community health workers to reach remote and underserved populations is crucial. Enhancing healthcare provider capacity through training and capacity building, fostering community ownership and engagement by leveraging the strengths of Somaliland's context, and strengthening data collection and monitoring systems are all essential components.

This research emphasises the crucial role of education and economic status in shaping women's ability to access healthcare services. Lower levels of education and lower wealth indices are associated with higher barriers to healthcare access, indicating the need for targeted interventions to address disparities. The study also underlines the impact of regional disparities, with women in certain regions facing more significant challenges in accessing healthcare compared to others. These disparities emphasise the importance of tailored healthcare strategies that consider the unique contexts and needs of different regions within Somaliland.

Furthermore, the study's multilevel analysis has provided valuable insights into the complicated net of factors influencing healthcare access among reproductive-age women. Factors such as maternal age, husband's employment status, contraceptive practices, and number of children born have emerged as significant predictors of healthcare access barriers. The findings highlight the need for comprehensive strategies that address not only individual-level factors but also community and regional underlying forces to ensure equitable access to healthcare services for all women in Somaliland.

Overall, this research highlights the crucial need for targeted interventions and policy measures to address the identified barriers to healthcare access among reproductive-age women in Somaliland. By understanding the complex web of factors influencing healthcare access, policymakers and healthcare providers can design interventions that are tailored to the specific needs of women in different regions and socioeconomic contexts. Improving healthcare access for women is not just a matter of improving health outcomes but also a crucial step towards achieving health equity and promoting the well-being of vulnerable populations in Somaliland.

## Data Availability

The datasets presented in this study can be found in online repositories. The names of the repository/repositories and accession number(s) can be found below: https://microdata.nbs.gov.so/index.php/catalog/50.
